# Modeling ecological traps for the control of feral pigs

**DOI:** 10.1002/ece3.1489

**Published:** 2015-04-22

**Authors:** Nick Dexter, Steven R McLeod

**Affiliations:** 1 Booderee National ParkVillage Rd, Jervis Bay, Jervis Bay Territory, 2540, Australia; 2 Vertebrate Pest Research Unit, NSW Department of Primary Industries, Orange Agricultural InstituteForest Road, Orange, New South Wales, 2800, Australia

**Keywords:** Attractive sink, control strategy, feral pigs, landscape scale pest control, predator–prey models, *Sus scrofa*

## Abstract

Ecological traps are habitat sinks that are preferred by dispersing animals but have higher mortality or reduced fecundity compared to source habitats. Theory suggests that if mortality rates are sufficiently high, then ecological traps can result in extinction. An ecological trap may be created when pest animals are controlled in one area, but not in another area of equal habitat quality, and when there is density-dependent immigration from the high-density uncontrolled area to the low-density controlled area. We used a logistic population model to explore how varying the proportion of habitat controlled, control mortality rate, and strength of density-dependent immigration for feral pigs could affect the long-term population abundance and time to extinction. Increasing control mortality, the proportion of habitat controlled and the strength of density-dependent immigration decreased abundance both within and outside the area controlled. At higher levels of these parameters, extinction was achieved for feral pigs. We extended the analysis with a more complex stochastic, interactive model of feral pig dynamics in the Australian rangelands to examine how the same variables as the logistic model affected long-term abundance in the controlled and uncontrolled area and time to extinction. Compared to the logistic model of feral pig dynamics, the stochastic interactive model predicted lower abundances and extinction at lower control mortalities and proportions of habitat controlled. To improve the realism of the stochastic interactive model, we substituted fixed mortality rates with a density-dependent control mortality function, empirically derived from helicopter shooting exercises in Australia. Compared to the stochastic interactive model with fixed mortality rates, the model with the density-dependent control mortality function did not predict as substantial decline in abundance in controlled or uncontrolled areas or extinction for any combination of variables. These models demonstrate that pest eradication is theoretically possible without the pest being controlled throughout its range because of density-dependent immigration into the area controlled. The stronger the density-dependent immigration, the better the overall control in controlled and uncontrolled habitat combined. However, the stronger the density-dependent immigration, the poorer the control in the area controlled. For feral pigs, incorporating environmental stochasticity improves the prospects for eradication, but adding a realistic density-dependent control function eliminates these prospects.

## Introduction

Density-dependent models of habitat selection, such as the ideal free distribution, have been very useful in explaining patterns of distribution and abundance in animals (Fretwell and Lucas 1970). These theories are based on the idea that habitats differ in carrying capacity and that the fitness of individuals within a habitat depends on population density so that density-dependent dispersal equalizes fitness between habitats varying in carrying capacity (McPeek and Holt 1992). When two habitats of equal carrying capacity are compared, the quality of one habitat can be improved for surviving individuals by lowering the density of individuals through culling, control, or harvest. Thus, there should be net immigration into the habitat in which density has been lowered as *per capita* resources will be higher and the habitat more attractive. However, fitness will be lower in the habitat where abundance has been controlled. If control mortality outweighs the benefits of lowered intraspecific competition, then an “ecological trap” has been created in the controlled habitat. Ecological traps are habitats where reproduction or survival cannot sustain a population, but this habitat is still preferred over other available higher-quality habitats that can sustain a population (Battin 2004). Normally, such fitness-reducing behavior will be selected against and most ecological traps have been described for situations where recent anthropogenic change has created a new poor quality preferred habitat (reviewed in Battin 2004). Control of a pest species throughout its range is considered a precondition for successful eradication (Bomford and O'Brien 1995). Due to the constraints of tenure, logistics, and finance, it is rare that a pest animal can be controlled throughout its entire range, thus dividing the landscape into uncontrolled sources and controlled sinks, assuming control is not biased toward habitat of a particular quality. Based on the results of models, ecological traps can cause the extinction of a population when the size of the trap exceeds some threshold (Delibes et al. 2001; Donovan and Thompson 2001; Kokko and Sutherland 2001; Kristan 2003). The significance of this to control efforts is that a pest may not need to be controlled throughout its entire range to be eradicated if the control area is an ecological trap.

In the current study, we modeled an ecological trap for feral pigs using a single-species logistic population growth model in which a pest population is divided into a controlled and an uncontrolled habitat with a density-dependent immigration rate, so that animals moved from high-density uncontrolled habitat to low-density controlled habitat. We examined the influence of three variables on the outcomes of this model.

The variables were as follows:

1. Control mortality: Of critical importance in successfully controlling a pest is the control mortality (Hone 1999). To successfully prevent a pest population from increasing, the rate at which pests are removed must be equal to or greater than the species' instantaneous annual rate of increase *r*_*m*_ (Hone 1999).
2. The proportion of total available habitat controlled: We divided the landscape into two patches – one in which pests are not controlled (the source) and one in which pests are controlled (the sink).
3. Strength of density-dependent immigration: While density-dependent habitat selection is common in mammals, it is not universal and may vary in strength within different populations of individual species (Matthysen 2005). To account for this, we modeled two scenarios of weak and strong density-dependent immigration.

Abrams et al. (2012) cautioned that the results of models of ecological traps may not hold for more complex multispecies models. Therefore, we built a second model using an existing empirically derived multispecies, stochastic, interactive model of feral pig population dynamics in the Australian rangelands (Choquenot 1998). We varied variables 1, 2, and 3 to examine how consumer–resource interactions alter the results compared with the logistic model. This model incorporated environmental stochasticity by linking population growth in feral pigs and kangaroos to prevailing pasture biomass, which was in turn linked to erratic rainfall.

Despite the simple logic of density-dependent ecological traps, the behavioral responses of the animals being controlled could alter the dynamics of ecological traps. For example, persistent control through hunting as opposed to poisoning or trapping could drive immigration from the controlled area to the uncontrolled area (Tolon et al. 2009). Therefore, to improve the realism of these models, we included a function describing the effect of declining pest density on control mortality. Successful pest control programs usually result in a decline in abundance of the pest, but as abundance falls, the effort required to remove further animals usually increases (Hone 1994, 2007). This can be due to the pest responding behaviorally to control and becoming less easy to manage as density decreases due to such things as acquired aversion to poison baits or traps (Caley and Ottley 1995; Morgan et al. 1996). Hone (1994) suggested that the decline in kill rate observed in pest control programs was equivalent to the functional response of predators to prey, which relates the per capita predation rate to prey density. Choquenot et al. (1999) used this approach to model the functional response of kill rate to pest density using data from three helicopter shooting programs for feral pigs. We incorporated these functional responses into the interactive model of feral pig population dynamics in place of fixed mortality rates to see whether this altered the outcomes from this model.

## Materials and Methods

### Logistic model

Our first model describes a population of pests growing logistically prior to any control. In this model, population size *N* is assumed to be proportional to the total area occupied by the population (*A*), with *A* limited and constant and where *r* is the intrinsic rate of increase. Therefore, in *A,* population growth is described by:

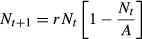
1

This population is then divided into two spatially discrete populations: an uncontrolled population (*Nu*) of pests and a controlled population of pests (*Nc*), linked by a density-dependent immigration function *f*(*G*,*γ*), in a contiguous area of otherwise similar habitat quality,

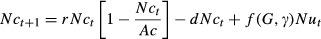
2and in the uncontrolled area (*Au*), population growth is described by:

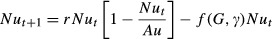
3

In these equations, *Nc*_*t*_ is the population size in *Ac* and *Nu*_*t*_ is the population size in *Au*. As habitat quality is constant and population size proportional to area available, the parameters *Ac* and *Au* are equivalent to carrying capacity in other versions of the logistic model and thus are measured in units of animals rather than area. The mortality rate due to control is *d*.

When *d* - 0, per capita resources are equal for *Nu*_*t*_ and *Nc*_*t*_. When *d* > 0, per capita resources are higher in *Ac* and individuals move from *Au* to *Ac* while the difference between the habitats remains. This creates an ecological trap in *Ac*. We assume control rate can be varied by increasing the frequency or intensity of effort. The density-dependent immigration rate function *f(G*_*t*_*,γ)* depends on *G*_*t*_, the ratio of per capita resources between *Au* and *Ac* (eq. 3), where *G*_*t*_ is the difference in the ratio of carrying capacity to population size between controlled and uncontrolled population:

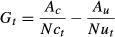
4

**Figure 1:**
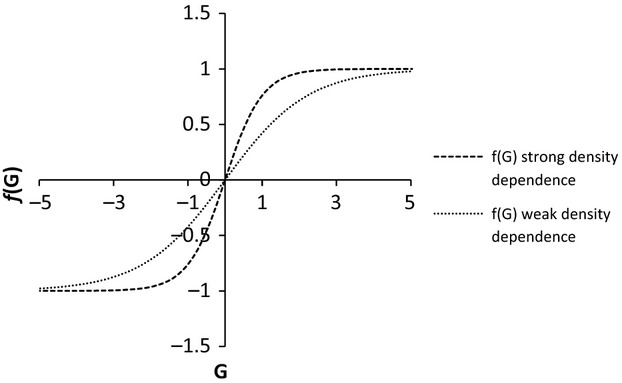
Relationship between difference in the ratio of carrying capacity to population size between controlled and uncontrolled population; *G*, and immigration rate; *f*(*G*).

When *A* is high relative to *N,* then the quotient of carrying capacity over population size approaches ∞. Immigration rate, *f(G*_*t*_*,γ)*, is modeled using the tanh function, so that:

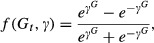
5

giving rise to a hyperbolic relationship between *f(G*_*t*_*,γ)* and *G*_*t*_ with the parameter *γ* controlling the strength of density-dependent immigration. Low values of *γ* give weaker density dependence and high values of *γ* give stronger density dependence (Fig.1). We could find no estimates of density-dependent immigration rates for feral pigs or wild boar to corroborate the veracity of this function.

The variables *Ac* and *d* are under the control of pest managers, so we systematically varied these parameters (*Ac*: 10%, 20%, 30%, 40%, 50%, 60%, 70%, 80%, 90% of the total area, and *d*: 10%, 50%, 90%). We chose feral pigs as a widely distributed mammalian pest that had evidence of density-dependent immigration (Hanson et al. 2009) and well-developed population models and an intermediate *r*_*m*_ (0.792) (Choquenot 1998). To examine variation in the strength of the density-dependent immigration, we modeled two scenarios for each combination of *Ac*, *d*, and *r*_*m*_, one with weak density depende-nce (*γ* - 0.1) and one with strong density dependence (*γ* - 1.0). Using a starting total population size of 1000 (*Nc*_*0*_ + *Nu*_*0*_), we recalculated equations 1 and 2 at 1-month time intervals for a 50-year sequence with *d* converted to a monthly control mortality rate. We chose 50 years as an ambitious but not impossible period of sustained pest control. A monthly rate was chosen because this is more likely to represent the relatively rapid dynamics of animal's responses to short-term changes in density and allow for smoother transitions between *Nu*_*t*_ and *Nc*_*t*_ over time. We recorded the mean population size of *Nc*_*t*_ and *Nu*_*t*_ and the time in months to extinction. Extinction was defined as less than two individuals in the combined population size of *Nc*_*t*_ + *Nu*_*t*_.

### Interactive model

We adapted a model of feral pig and red kangaroo *Macropus rufus* Desmarest dynamics in the semi-arid rangelands of Australia (Choquenot 1998) to explore whether the conclusions of the logistic model also applied to more complex models when environmental stochasticity was included. The model we chose has been extensively used to explore the ecology and management of feral pigs in Australian rangelands (Choquenot and Ruscoe 2003; Dexter 2003).

The three components of this model system are change in vegetation biomass, change in kangaroo abundance, and change in pig abundance. The system changes quarterly (90 days) and is described by the equations:

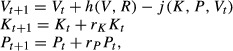
6

where *V*_*t*_ is vegetation biomass (kg ha^−1^) at time *t*, *K*_*t*_ and *P*_*t*_ are the abundance of kangaroos and pigs (number km^−2^), respectively, *h(V, R)* is the growth in vegetation biomass, *j(K, P, V*_*t*_*)* is the consumption of vegetation by kangaroos (*K*) and pigs (*P*), and the variables *r*_*k*_ and *r*_*P*_ are the rate of increase of kangaroos and pigs, respectively.

The function *h(V, R)* takes the form:

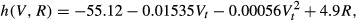
7 where *R* is the rainfall in the current quarter.

The function *j(K, P, V*_*t*_*)* combines the functional respo-nses of kangaroos and pigs and assumes that there is no interference between the herbivores. It takes the form:

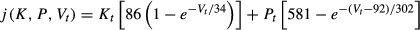
8

The functional response of red kangaroos was estimated by Short (1987), while the functional response of pigs was estimated by Choquenot (1998). The numerical response equations describing the yearly change in herbivore abundance (*r*_*K*_ for kangaroos and *r*_*P*_ for pigs) were estimated by Caughley (1987) for kangaroos and Choquenot (1998) for pigs. These equations take the form:

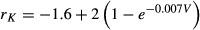
9

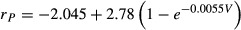
10

Annual rates of increase were converted to quarterly increments by dividing equations 8 and 9 by four.

The interactive model of feral pig dynamics was converted to a source–sink model by the inclusion of a density-dependent immigration function *f(G*_*P*_*,γ)* to link an area that had feral pig control (*Pc*) to an area where feral pigs were not controlled (*Pu*):

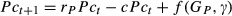
11



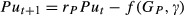
12 Density-dependent immigration was calculated by the variable *G*_*p*_:

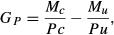
13

where *M*_*c*_ was the number of pigs that would be expected to be present in the controlled area if there was no control, no density-dependent immigration, and conditions were identical to those experienced in area *Pc*. Similarly, *M*_*u*_ was the number of pigs expected to be present in the uncontrolled area if there was no control, no density-dependent emigration, and conditions were identical to those experienced in area *Pu*. The function *f(G*_*P*_*,γ)* was calculated in the same manner as *f*(*G γ*) (eq. 4) using the tanh function. Based on the data of densities of feral pigs and kangaroos in Australian rangeland, simulations were initialized using 4 pigs km^−2^ (Choquenot et al. 1999) and 45 kangaroos km^−2^ (Caughley 1987) and with an initial vegetation biomass of 295 kg ha^−1^. The initial value for the variable *Pc* was calculated by multiplying the initial density of pigs by the proportion of the total area that the controlled area represented (equivalent to *Ac* in eq. 1) by 1000. The value of 1000 was chosen as this represents the approximate area in km^2^ of the Paroo River system in semi-arid Australia, where the parameters for this study were estimated and control of feral pig populations is regularly conducted. Kangaroos were not harvested and hence there was no density-dependent immigration. For each quarter of a 50-year simulation, rainfall (*R*) was a random draw from the distribution of quarterly rainfall recorded in Wanaaring in semi-arid Australia (mean 193 mm, standard deviation 90 mm) (Dexter 2003). However, the model was run at a monthly time step with all equations converted to a monthly time step for a 50-year sequence. The parameters *Mc* and *Mu* were calculated by simultaneously running a model with the same random draw of rainfall but without c or *G*_*P*_ in equations 10 and 11. We recorded the mean population size of *Pc* and *Pu* and the time in years to extinction for 1000 runs of each possible combination of *Pc* (10%, 20%, 30%, 40%, 50%, 60%, 70%, 80%, 90%), *c* (10%, 50%, 90%), and *γ* (0.1, 1.0). Extinction was defined as there being less than two individuals in the combined population size of *Pc* + *Pu*.

### Interactive model with density-dependent control rate

The effort required to control a pest often increases as pest density decreases (Hone 1994). To model control effort as a function of density, we replaced the fixed control rate parameter *c* with the function *q(P)*, which describes the change in control efficiency with changing pest density. Choquenot et al. (1999) describe the derivation of this function for feral pigs shot from helicopters, for three widely separated study sites in Australia.

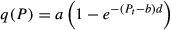
14

**Figure 2:**
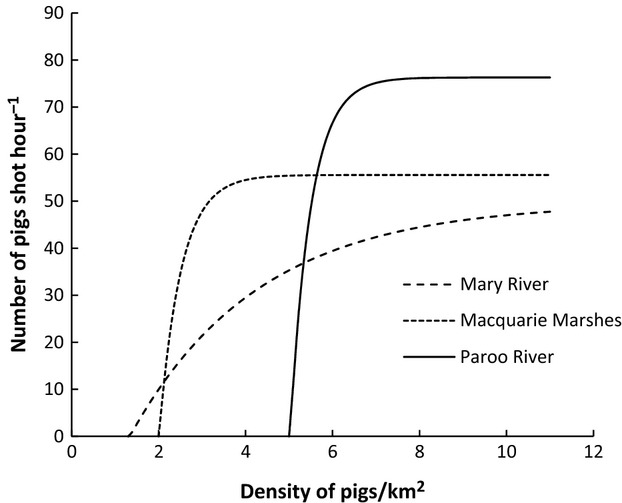
Functional response models fitted to the relationship between kills h^−1^ and pigs km^−2^ for helicopter shooting programs for feral pigs, conducted on the Mary River, Macquarie Marshes, and Paroo River.



This functional response – measured in pigs killed hour^−1^ (Fig.2) – is characterized by three parameters: *a* the maximum kill rate above some threshold pig density (which is equivalent to a predator's saturated rate of prey off-take measured in pigs killed hour^−1^), *d* the efficiency of the shooting program (which is equivalent to the relative effect that declining prey density has on the rate of prey off-take), and *b* the predicted density below which no more pigs can be shot (which is equivalent to the existence of a prey refuge). We incorporated this equation into our model by modifying equation 10 to give:

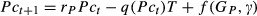
15



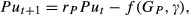
16where *T* is the total time spent shooting, and the other parameters retain their previous definition. When *Pc*_t_ was below *b,* the function *q*(*P*c_t_) was curtailed at 0 so that no pigs were shot. The values used for the parameters are listed in Table1.

**Table 1:** Parameters for equation 13, the functional response kills h^−1^, and pigs km^−2^ for helicopter shooting programs for feral pigs

Study area	*a*	*b*	*d*
Mary River	49.643	1.338	0.339
Macquarie Marshes	55.552	2.008	1.986
Paroo River	76.282	5.023	2.115

All other parameters, time steps, and definition of extinction were the same as those defined for equations 12 and 13. To convert equation 13 – an hourly rate – to the time taken for a shooting exercise, we multiplied *q*(*Pc*_*t*_) by 7.5 (7.5 h being the approximate time taken to search and shoot 1000 km^2^ (Choquenot et al. 1999). We assumed there was one shooting session of 7.5 h per month. While the parameters *a*, *b*, and *d* are different for each of the three sites (Table1), we only had a parameterized population model for the Paroo River site and assumed that the population dynamics of feral pigs were similar on the other sites. Therefore, we ran three versions of the stochastic interactive model using the parameterized population model for the Paroo River site with the three different versions of *q*(*Pc*_*t*_) for the three sites**.** For each of the three versions of *q*(*Pc*_*t*_), we recorded the mean population size of *Pc* and *Pu* and the time in years to extinction for 1000 runs of each possible combination of *Pc* and *γ*. Extinction was defined as there being less than two individuals in the combined population size of *Pc* + *Pu*. All models were constructed in an EXCEL® spreadsheet.

## Results

### Logistic model

**Figure 3:**
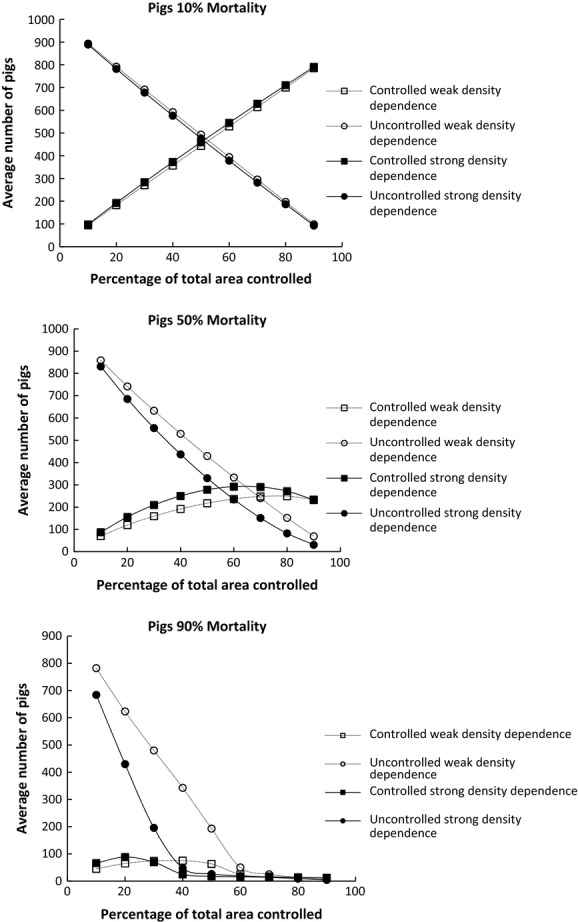
Relationship between abundance and proportion of area controlled for controlled area and uncontrolled area with three annual control mortality rates for feral pigs using the logistic model.

The long-term average densities of controlled and uncontrolled areas from the simulations are shown in Figure3. These results show two important findings. The first is that increasing both control mortality and proportion of area controlled caused a decrease in abundance in both the controlled and uncontrolled habitat and increased the probability of extinction in these simulations (Fig.3 and Table2). The second important finding is that increasing the strength of density-dependent immigration caused the control area to have relatively higher pest abundance and the uncontrolled area relatively lower pest abundance, if all other parameters were kept the same in these simulations (Fig.3). Thus, when density-dependent immigration is strong, below a certain control mortality and proportion of an area controlled, any benefit from control is likely to be lost in the control area because it will rapidly be swamped by immigrants from the uncontrolled area. Conversely, for populations with weak density dependence, lower population densities can be more easily maintained in the controlled areas but there will be less effective control in the uncontrolled areas because there will be less emigration to the controlled area.

**Table 2:** Time in years to extinction for feral pigs using the logistic model

Proportion of area controlled	Pig; 90% control rate, weak density dependence	Pig; 90% control rate, strong density dependence
10%		
20%		
30%		
40%		47.3
50%		22.7
60%	32.6	20.9
70%	23.5	19.4
80%	19.3	17.5
90%	15.3	14.3

### Interactive model

**Figure 4:**
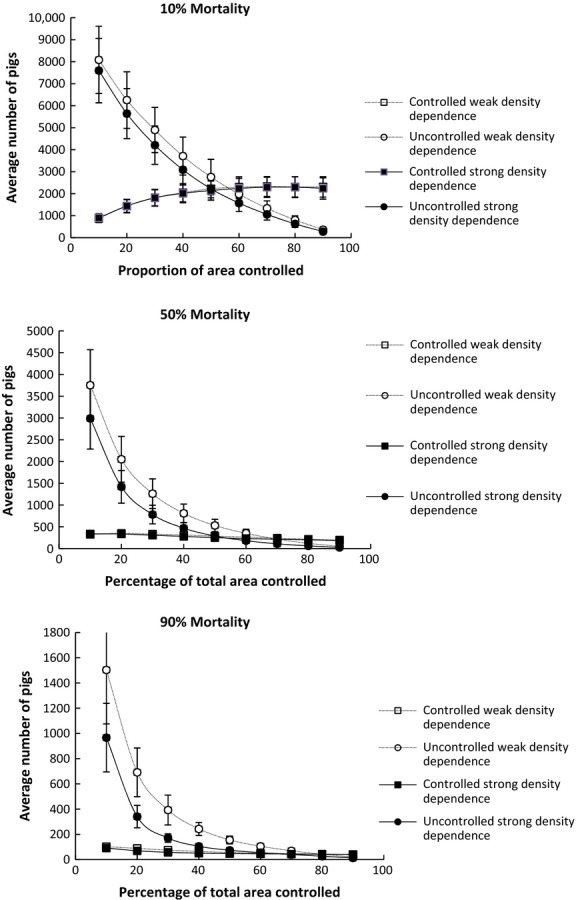
Relationship between average abundance (±1SD) and proportion of area controlled for controlled area and uncontrolled areas with three annual control mortality rates for feral pigs using the interactive model.

The results of simulations using the interactive model of feral pig dynamics were broadly similar to the conclusions drawn from the results of the logistic models. Increasing the proportion of habitat controlled, control mortality, and the strength of density-dependent immigration all increased the probability of extinction and lowered overall density (Fig.4 and Table3). However, two differences in outcomes are apparent. First, the time to extinction was lower for any given value of proportion of habitat controlled, control mortality, or strength of density dependence than the logistic model (Table3). Second, the effect of increasing the strength of density-dependent immigration was much weaker than for the logistic model with there being little difference in abundance for controlled areas between weak and strong density dependence simulations. However, substantial differences in abundance remained between weak and strong density dependence simulations for uncontrolled areas.

**Table 3:** Average time in years and standard deviation in years to extinction for feral pigs using the interactive model

Proportion of area controlled	Weak density dependence, 50% control rate and SD	Strong density dependence, 50% control rate and SD	Weak density dependence, 90% control rate and SD	Strong density dependence, 90% control rate and SD
10%			37.2 (4.7)	34.1 (5.5)
20%	49.2 (1.9)	47.3 (3.4)	22.3 (2.8)	19.0 (2.7)
30%	41.1 (3.6)	36.8 (3.8)	16.7 (2.3)	13.6 (1.8)
40%	33.1 (3.0)	29.3 (2.9)	13.2 (1.3)	10.8 (1.2)
50%	28.2 (2.9)	25.3 (2.7)	11.3 (0.9)	9.4 (0.8)
60%	23.5 (2.2)	20.9 (2.0)	10.2 (0.7)	8.8 (0.6)
70%	20.1 (1.7)	18.0 (1.6)	9.4 (0.6)	8.5 (0.5)
80%	17.3 (1.4)	15.8 (1.3)	8.8 (0.6)	8.1 (0.6)
90%	14.9 (1.1)	14.0 (1.0)	7.9 (0.6)	7.4 (0.5)

### Interactive model with density-dependent control rate

**Figure 5:**
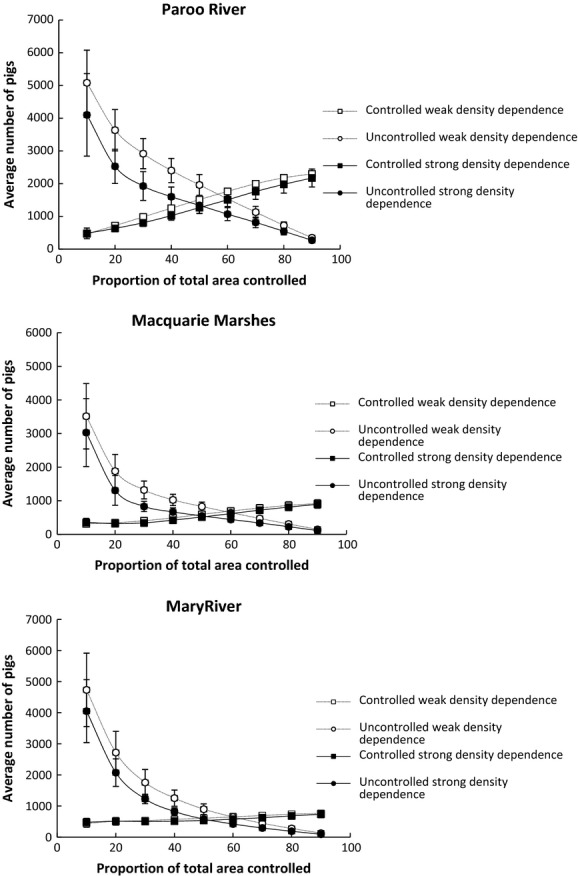
Relationship between average abundance (±1SD) and proportion of area controlled for three density-dependent control mortality rates for three populations of feral pigs using the interactive model with density-dependent control.

The results of the simulations can be seen in Figure5. The chief difference with the interactive model with fixed control rates is that no populations went extinct although populations in the Macquarie Marshes and Mary River were driven to very low levels of abundance. Further, the density-dependent constraints on control mortality ensure that there is little decrease in abundance with increasing the proportion of the area controlled as abundance asymptotically approaches a stable level. Despite the fact that the highest killing rate of feral pigs could be achieved in the Paroo River region, it also had the highest average density of pigs following control, due to having the lowest shooting efficiency and the highest threshold below which no pigs could be shot. In contrast, in the Macquarie Marshes and Mary River regions, the combination of higher shooting efficiencies and lower thresholds resulted in lower overall abundances following control.

## Discussion

### Logistic model

Our results suggest that theoretically increasing both the area controlled and control mortality lowers overall pest abundance and increases the probability of extinction for feral pigs. These results are in line with other ecological trap models (Delibes et al. 2001; Abrams et al. 2012) that predict extinction will be achieved when a certain threshold proportion of habitat is controlled.

In our models, the strength of density dependence was speculative. However, density-dependent habitat selection is a common phenomenon (Travis et al. 1999) having been detected in a wide variety of mammals (Morris 1987; Ovadia and Abramsky 1995; Edwards et al. 2002; Shenbrot 2004). Among territorial pest species with an ideal despotic form of habitat selection, density dependence is likely to be particularly strong as not only will dispersing individuals be attracted to the ecological trap because of higher per capita resources but also they will be pushed toward this habitat by dominant territory holders in the uncontrolled habitat. In a study of the culpeo fox, *Pseudalopex culpaeus* Molina, researchers divided habitat into sheep ranches, where foxes were persecuted and at low density, and cattle ranches, where foxes were not persecuted and at high density (Novaro et al. 2005). Radio-collared foxes consistently moved toward the less densely populated and more dangerous sheep ranches where they suffered a higher mortality rate. For red foxes, Baker et al. (2000) described the process as extending existing territorial boundaries until neighboring dominant animals were encountered. Similarly, for common brushtail possums, *Trichosurus vulpecula* Kerr, in New Zealand, expansion of home ranges into areas subject to control has been observed (Efford et al. 2000; Pech et al. 2010). Even nonterritorial pest mammals, such as feral horses, *Equus ferus caballus* Linnaeus, have been shown to have density-dependent immigration (Berger 1987), and there is strong indirect evidence for density-dependent immigration by feral pigs into intensively hunted areas from less intensively hunted areas (Hanson et al. 2009).

In the logistic models, varying the strength of density dependence had less impact on the outcomes than varying control mortality or proportion of habitat controlled. Nonetheless, the outcomes for the logistic model illustrated an interesting phenomenon. For simulations with weak density-dependent immigration, control will be more efficient at lowering abundance in the controlled area than for simulations with stronger density-dependent immigration. When immigration is weakly density dependent, the controlled area will be replenished from uncontrolled areas at a lower rate than for high-density-dependent situations. This means that at a local scale, control is likely to be more successful for pest species with weak density-dependent immigration than for species with strong density-dependent immigration, but at the landscape level of controlled and uncontrolled habitat combined, control will be more successful for species with strong density dependence.

Our results suggest that small geographic-scale control operations maybe ineffective if density-dependent immigration “swamps” control efforts but that large-scale operations will have benefits well beyond the area being controlled. The importance of the spatial scale of control is illustrated by comparing the results of two experimental studies of fox control in Australia (Greentree et al. 2000, Dexter and Murray 2009). In the study by Greentree et al. (2000) fox control sites were hundreds of hectares, but there was no significant impact of fox control on fox abundance within these control sites. In contrast, in the study by Dexter and Murray (2009), where treatment sites were >10,000 hectares, there was a negative effect of control on fox abundance well beyond the boundaries of the control sites.

### Interactive model

Compared to the logistic model of feral pig control, the interactive model predicted extinction at a lower proportion of area controlled and lower control mortality. This conforms to the widely accepted belief that populations inhabiting environments of greater resource fluctuation will be more prone to overharvest than populations in more stable environments (Lande et al. 2003). Under the conditions of the stochastic interactive model, the differences in outcomes between weak and strong density-dependent immigration were less than for the logistic model. This may represent the overriding importance of the interaction between a highly variable rate of increase and the mortality rate due to control in this model. While the interactive model is considerably more complex than the logistic model, many feral pig populations occur in stable environments such as temperate or tropical forests (Singer et al. 1981; Mitchell et al. 2007) where the logistic model may be a better approximation of population processes.

Considering the results of the interactive model broadly, pest species such as mustelids, lagomorphs, and rodents are clustered at the higher end of the *r*_*m*_ spectrum (Duncan et al. 2007). The results of the logistic model for feral pigs suggest that the benefits of creating ecological traps may be limited for controlling species with much higher rates of increase. However, the results of the stochastic interactive model suggest more optimistic prospects for controlling these species. This is because many populations of these species including house mice (Brown and Singleton 1999), European rabbits (Wood 1980), stoats (*Mustela erminea* Linnaeus), and weasels (*Mustela nivalis* Linnaeus) (Korpimaki et al. 1991) are subject to population fluctuations of much greater magnitude than feral pigs, and thus, the effort required to achieve effective control will be underestimated by the logistic model.

The prospects for eradication and high levels of control were not supported when density-dependent control rates were included. In our simulations, parameter *b* defined a population density boundary below which control mortality was zero while *a* defined an upper boundary that prevented effective control at high densities. Variation in the control parameter values for the three study sites yielded different mean abundances for the same population parameters (derived from the Paroo River population). However, the overall patterns are roughly similar with an initial steep decline in abundance – but with a decreasing rate of decline – with increasing proportion of area controlled.

Different control techniques are likely to yield different functions describing the change in control efficiency with changing pest density (Hone 1994). Poisoning can reduce feral pig abundance by 83% (Choquenot et al. 1993), but uptake of poison bate is dependent on the availability of alternative food (Choquenot and Lukins 1996). Thus, increasing *per capita* resources with decreasing abundance in controlled areas may lead to reduced bait take. The relationship between control rate and the presence of ecological traps may be further complicated if the pest species changes activity in response to the risk of control mortality, with the ecological trap becoming the less preferred habitat. Wild boar (*Sus scrofa* Linnaeus) will select protected areas in preference to nonprotected areas where the risk of hunting mortality is higher (Tolon et al. 2009). However, several studies (Dexter 1996; Campbell et al. 2010) found that feral pigs did not change movement patterns in relation to helicopter shooting. This difference between the response of wild boar to hunting and of feral pigs to helicopter shooting may be due to the length of time that each control activity has been in action; hunting is usually conducted in an area over a long time period while helicopter shooting is typically sporadic. This means that persistent hunting by hunters on foot with dogs may drive pigs out of controlled areas into uncontrolled areas.

## Conclusion

The logic of ecological traps has been applied to conservation biology (Battin 2004) and fisheries management (Shepherd and Litvak 2004; Kellner et al. 2008; Abrams et al. 2012) but not pest control. The models we present show that with increasing control mortality and increasing proportion of the habitat controlled, there could be benefits at a landscape scale both within the controlled and uncontrolled areas. However, below certain levels of control and proportion of an area controlled, any benefit from control is likely to be lost because of immigration into the control area.

The phenomena we highlight are of particular pertinence to eradication attempts. Bomford and O'Brien (1995) described six preconditions for pest eradication, one of which is that immigration is prevented. As this precondition is very rarely achievable except on small islands, it renders all attempts at eradication unlikely a priori. The results of this study suggest this precondition is not valid. Indeed, density-dependent immigration can be used more broadly to reduce the abundance of pests from a larger area than the area under control if a sufficiently high control rate can be maintained for species with relatively low rates of increase. Realistically, it is easier to manipulate the amount of area controlled as this can be predetermined exactly whereas a particular control effort cannot guarantee a particular control mortality, as demonstrated by Choquenot et al. (1999). We believe that control programs could readily be designed to test the power of density-dependent immigration to improve outcomes by measuring densities, and immigration rates (using telemetry) across a range of control rates and proportions of area controlled. These models did not investigate the spatial dispersion of harvested and unharvested areas on overall mortality rates, and it may be that, as with some metapopulation models, dispersion of patches (King and With 2002) or landscape texture (Gamarra 2005) may alter some of the predictions of this model. This kind of habitat heterogeneity along with behavioral and ecological heterogeneity should be the focus of further theoretical and empirical investigations into the applications of ecological traps to pest control.
